# Learning and teaching ophthalmology in the pandemic

**DOI:** 10.31744/einstein_journal/2022CE6988

**Published:** 2022-06-01

**Authors:** Thiago Gonçalves dos Santos Martins, Gabriel Lima Benchimol, Gustavo Rosa Gameiro, Paulo Schor

**Affiliations:** 1 Universidade Federal de São Paulo São Paulo SP Brazil Universidade Federal de São Paulo, São Paulo, SP, Brazil.; 2 Universidade Estácio de Sá Rio de Janeiro RJ Brazil Universidade Estácio de Sá, Rio de Janeiro, RJ, Brazil.; 3 Hospital Israelita Albert Einstein Faculdade Israelita de Ciências da Saúde Albert Einstein São Paulo SP Brazil Faculdade Israelita de Ciências da Saúde Albert Einstein, Hospital Israelita Albert Einstein, São Paulo, SP, Brazil.

Dear Editor,

The coronavirus disease 2019 (COVID-19) pandemic had a major impact on medical education, which had to be redesigned to use new platforms and teaching resources to complement the traditional in-person modality.^(1)^ Ophthalmology was one of the specialties with more residents infected by coronavirus in New York City. This was due to the proximity between the physician and patient during the ophthalmologic examination.^(2)^ Therefore, teaching of ophthalmology had to be adjusted for undergraduate medical students.

Teaching of direct ophthalmoscopy has been reduced in the syllabus of medical schools, thus increasing lack of confidence of physicians when dealing with basic ophthalmological problems, and hindering the appropriate triage of the population for eye diseases.^(3)^ In 2006, the International Council of Ophthalmology established the minimum knowledge on ophthalmology expected from undergraduate medical students would include the red reflex testing and appropriate direct ophthalmoscopy examination. This would enable satisfactory training in this field, which accounts for approximately 5% of all medical urgences.^(4)^

Hence, trying to improve quality when teaching an examination technique, which had always been briefly addressed in the medical syllabus and was even more hindered during COVID-19 pandemic, due to social distancing, we have developed a new teaching method. It was employed for the seventh-semester undergraduate medical students at *Faculdade Israelita de Ciências da Saúde Albert Einstein* (FICSAE), to teach red reflex testing and appropriate direct ophthalmoscopy examination.

This new methodology provided theoretical teaching by using free and open platforms, such as YouTube, in which a tutorial was given to undergraduates, who were able to build a teaching dummy that enabled understanding the fundamental principles of physics for the direct ophthalmoscopy examination. Moreover, this could rise the students´ interest and participation, even before the in-person classes.^(5)^ Online theoretical classes were given before the practical classes, reducing the duration of in-person classes, and not affecting quality of teaching.

**Figure 1:**
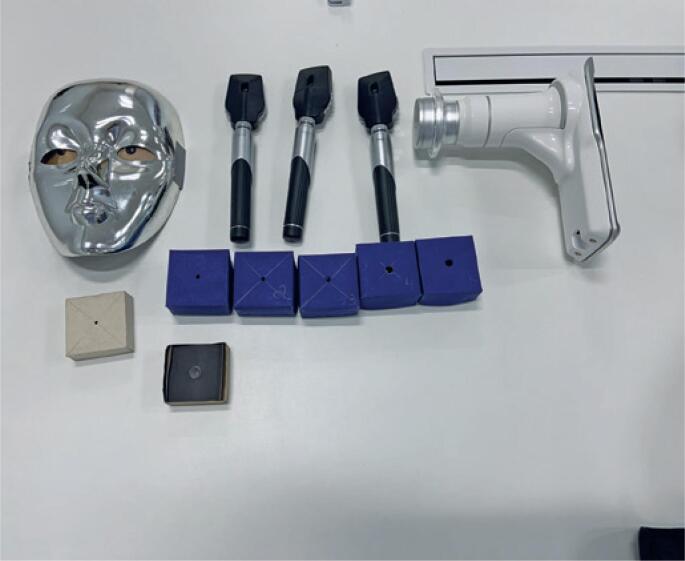
Dummy and portable handheld fundus camera to teach red reflex testing and direct ophthalmoscopy

During the practical classes, teaching dummies and a portable handheld fundus camera were used, and the undergraduates were followed up in real time when performing direct ophthalmoscopy examination (Figure 1). This method of teaching ophthalmoscopy based on dummies with pupils of different sizes allowed training with increasing difficulty levels, before teaching the technique to students. Thus, teaching with dummies and using portable handheld fundus cameras led to more interaction and better understanding among students, and it also decreased their training time. Even wearing N95 masks, their period of exposure was reduced.

Proper medical training has not kept abreast the growth of medical schools, and teaching and learning have been hindered during the social distancing period imposed by COVID-19 pandemic. Direct ophthalmoscopy examination enables diagnosis of diseases that may threaten the vision or life of patients, and its teaching has also been affected by this period. The use of new teaching methodologies that include use of technology, remote education and low-cost dummies, resulted in a more democratic teaching of direct ophthalmoscopy, besides reducing time for in-person training, and had no impact on quality of teaching of this important examination.
